# The Impact of Health Literacy on Consumer Knowledge of Mask and Hand Sanitizer Use in Post-Pandemic Korea

**DOI:** 10.3390/healthcare13020125

**Published:** 2025-01-10

**Authors:** Youngill Ko, Seung-Mi Lee

**Affiliations:** 1 College of Pharmacy, Daegu Catholic University, Gyeongsan 38430, Republic of Korea; pharmbig@naver.com; 2 The Gyeongbuk Pharmaceutical Association, Gumi 39280, Republic of Korea

**Keywords:** health literacy, masks, hand sanitizers, COVID-19 pandemic, public health

## Abstract

Background/Objectives: The COVID-19 pandemic increased people’s reliance on masks and hand sanitizers, highlighting the critical role of health literacy (HL) in effectively using these preventive measures. This study aimed to assess how HL influences consumers’ comprehension of product labels for masks and hand sanitizers in post-pandemic Korea. Methods: A cross-sectional online survey was conducted in September 2023 among 500 Korean adults aged 20–69. The participants completed a questionnaire measuring HL and their knowledge of mask and hand sanitizer labels using the Korean Health Literacy Assessment Tool. The respondents were classified into high- and low-literacy groups, and statistical analyses (chi-squared tests, *t*-tests, and logistic regression analyses) were employed to compare label comprehension between the groups. Results: High HL was observed in 31.6% of participants, demonstrating significantly better label-reading habits, including greater recognition of quasi-drug designations, usage instructions, and safety precautions (*p <* 0.001). The logistic regression analysis revealed that high-HL participants were more likely to correctly identify mask-related information (OR 2.56, 95% CI: 1.69–3.89) and demonstrate hand sanitizer-related knowledge (OR 5.39, 95% CI: 3.31–8.77) than their counterparts. Demographic factors such as age, gender, or education showed no significant associations with label comprehension. Conclusions: Higher HL is strongly associated with better comprehension of mask and hand sanitizer labels, underscoring its importance in public health communication. Enhancing HL is essential in promoting the safe use of preventive products during health emergencies. Public health initiatives should prioritize improving health literacy to ensure more effective communication and safer practices during future health crises.

## 1. Introduction

Coronavirus disease 2019 (COVID-19) has brought about significant changes in public health practices and regulations globally [1,2,3]. Among the various measures implemented to mitigate the spread of the virus, the utilization of masks and hand sanitizers has been paramount. These protective behaviors have become integral to daily life, underscoring the necessity of clear and effective communication regarding their proper use and effectiveness. Studies indicate that hand hygiene and mask-wearing behaviors vary significantly based on individuals’ knowledge and perceptions, highlighting the critical role of public health campaigns in promoting these measures [4,5]. Moreover, understanding how consumers interpret and utilize information on product labels, particularly for quasi-drug items such as masks and hand sanitizers, is crucial in ensuring the effectiveness of these interventions [6]. The accurate interpretation of labeling information is necessary to mitigate misuse; for example, the inappropriate use of hand sanitizers has led to health issues such as skin irritation and accidental ingestion, particularly among vulnerable populations [7,8]. Consequently, ensuring that label information is accessible and comprehensible is essential in maximizing the efficacy of these preventive measures.

Health literacy (HL), or the ability to acquire, interpret, and comprehend essential health information and services, is crucial in this context [9]. Previous studies have demonstrated that individuals with higher HL levels are better equipped to understand medical instructions, adhere to treatment protocols, and engage in preventive health behaviors [10]. HL extends beyond individual knowledge and is inextricably linked to the communication of healthcare information, emphasizing the importance of information that is clear and accessible [11]. Conversely, low HL is associated with poor health outcomes, the reduced utilization of preventive services, and increased hospitalization rates [12,13,14,15]. For instance, individuals with limited HL may have difficulties in interpreting medication labels, potentially leading to the improper use of nonprescription drugs and adverse health consequences [16,17]. These disparities highlight the need for improved strategies for the presentation of health information, ensuring that individuals with limited literacy levels can make informed health decisions.

Ensuring the correct usage of health-related products is critical in preventing the spread of infectious diseases. However, there is a limited understanding of the effectiveness with which consumers interpret and act upon the information presented on the labels of masks and hand sanitizers—products that are regulated as quasi-drugs in Korea. These items carry specific classification, usage, and safety instructions that consumers may find challenging to interpret, especially if their HL is low. While previous research has established a general relationship between HL and health outcomes, few studies have examined how HL specifically influences consumer engagement with quasi-drug labels.

Therefore, this study addresses a key gap in the literature, focusing on how HL impacts the comprehension and reading behaviors associated with these labels. Through an online survey of 500 participants, we investigated whether individuals with higher HL levels exhibited a greater understanding of quasi-drug classifications, usage instructions, and safety precautions compared to those with lower HL. We also examined how thoroughly consumers read product information based on their HL levels. Our objective was to provide evidence-based insights to inform public health communication strategies aimed at improving the clarity and accessibility of label information. These findings have important implications for Korea, where quasi-drug products are subject to specific cultural and regulatory conditions, as well as for other regions seeking to enhance the safe and effective use of infection prevention products. Ultimately, this research underscores the global relevance of health literacy in promoting compliance with safety guidelines and proper usage practices, particularly during public health emergencies.

## 2. Materials and Methods

### 2.1. Study Design and Participants

A cross-sectional design was used to investigate consumers’ knowledge and comprehension of mask and hand sanitizer use in the post-COVID-19 pandemic context. The study included participants aged 20 to 69 years so as to focus on the working-age population, who are more likely to actively engage with quasi-drug products such as masks and hand sanitizers. Individuals aged 70 and above were excluded due to their reduced tendency to use online platforms for surveys and potential differences in their health behaviors. The required sample size was determined based on a significance level (α) of 0.05, statistical power (1-β) of 80%, a proportion of 30% for high health literacy, and an estimated 20% proportion of individuals with high label comprehension, with the aim of detecting an odds ratio of at least 2 [18]. This calculation indicated the need for at least 433 participants to achieve sufficient power. To ensure a robust statistical analysis, we recruited a total of 500 participants.

The participants were recruited online through a reliable professional survey agency, using proportional quota sampling based on gender and age groups, according to the 2020 Korean Census data [19]. This agency had expertise in public opinion polling, surveys, and statistical analysis, with nearly 20 years of experience in conducting research, and it maintained an online research panel of over one million Koreans. From this extensive panel, adults aged 20–69, residing nationwide, were invited via text messages and emails to participate in the survey. Only those who responded to the survey through an online link were able to participate. Recruitment was carried out sequentially until the completed response quotas according to the gender and age groups were fulfilled. Once a quota was filled, no further recruitment was conducted. The recruitment of the participants and the online survey were conducted in September 2023.

### 2.2. Data Collection and Assessment

Data collection was conducted via a structured online questionnaire. The questionnaire included sections on demographic information, HL levels, and specific questions about the respondents’ knowledge and understanding of labels on masks and hand sanitizers. The demographic information included the participants’ gender, age, marital status, household size, education level, monthly income (USD), and employment status. HL was assessed using the Korean Health Literacy Assessment Tool (KHLAT), which is an adaptation of the Rapid Estimate of Adult Literacy in Medicine (REALM), designed for the Korean population [20,21]. Unlike the REALM, which evaluates health literacy based on the correct pronunciation of words, the KHLAT was specifically adapted for the Korean language, where pronunciation is not a meaningful proxy for understanding due to the nature of the written script. The participants were presented with a list of 66 health-related words and asked to indicate their level of understanding of each word by selecting one of the following options: “I know it well”, “I partially know what it means”, “I have heard of it, but do not know its meaning”, or “I do not know it”. Only words for which the participants selected “I know it well” were scored. The total score was calculated as the sum of such responses. The scores were then categorized as follows: individuals scoring 61 or more words were classified as having high HL, while those with scores below 61 were classified as having low HL.

Regarding mask and hand sanitizer labels, the questionnaire was divided into the following categories: indication of “quasi-drugs”, indications and effects, dosage and administration, all ingredients, usage precautions, storage methods, and expiration dates. The participants rated how thoroughly they read each section using a 5-point Likert scale. Five different items representing masks and five different items representing hand sanitizers—all registered with the Ministry of Food and Drug Safety and commercially available—were collected to develop questions assessing the comprehension of mask and hand sanitizer labels. The information displayed on the packaging of each product was systematically documented. The collected label information was then reviewed by two licensed pharmacists with experience in both pharmacy practice and research, who assigned priority rankings based on the perceived importance of each item. The highest-priority items were selected to construct the comprehension assessment. Specifically, 8 questions for mask labels and 10 questions for hand sanitizer labels were developed, focusing on key safety and usage information identified by the pharmacists. Those who demonstrated awareness of ≥80% of the items related to mask and hand sanitizer information were classified as “respondents with comprehensive knowledge”.

### 2.3. Ethical Considerations

This study was conducted according to the guidelines of the Declaration of Helsinki and the ethical standards of the institutional review board (IRB) of Daegu Catholic University, which approved the study protocol (approval number: CUIRB-2023-0033 and approval date: 18 August 2023). Informed consent was obtained from all participants before they completed the survey. They were assured of the confidentiality of their responses and informed of their right to withdraw from the study at any time without consequences.

### 2.4. Statistical Analysis

Descriptive statistics were employed to summarize the demographic characteristics of the study population, with the results stratified by gender to explore potential gender-based differences. Mean values and standard deviations were calculated for continuous variables, while frequencies and percentages were utilized for categorical variables. To validate the survey instrument, Cronbach’s alpha coefficient was calculated to assess the internal consistency of the items.

Comparative analyses were conducted using chi-squared tests and t-tests to examine the differences between the low- and high-HL groups regarding their label-reading habits and knowledge levels. Logistic regression analyses were performed to identify factors influencing the correct knowledge of mask and hand sanitizer use. Odds ratios (ORs) and 95% confidence intervals (CIs) were determined for these analyses.

All statistical analyses were performed using the SAS software (ver. 9.4, SAS Institute Inc., Cary, NC, USA), with the significance level set at *p* < 0.05.

## 3. Results

A total of 500 participants, comprising 254 men (50.8%) and 246 women (49.2%), were included in this study. The demographic characteristics of the study population are presented in Table 1. Regarding marital status, 42.2% were single and 57.8% were married. The household size data showed that 17.4% lived alone, 20.6% with one other person, and 62.0% in households of three or more. Regarding the participants’ educational levels, 18.6% had attended high school, 71.8% had attended university, and 9.6% had postgraduate qualifications. The data on their monthly income levels indicated that 14.4% earned below USD 722, 25.6% earned between USD 722 and 2166, 30.0% earned between USD 2167 and 3610, 20.8% earned between USD 3611 and 5055, and 9.2% earned USD 5056 or higher. Regarding employment status, 57.4% were in full-time employment, 11.0% were in part-time employment, and 31.6% were unemployed. There were significant gender-related differences in the employment statuses and income levels, with women more likely to be in part-time work or unemployed and reporting lower monthly incomes compared to men. However, despite these socioeconomic disparities, women demonstrated significantly higher HL scores than men. The internal consistency of the KHLAT, which comprises 66 health-related words, was evaluated using Cronbach’s alpha. The alpha coefficient ranged from 0.988 to 0.989, indicating excellent reliability.

**Figure 1:**
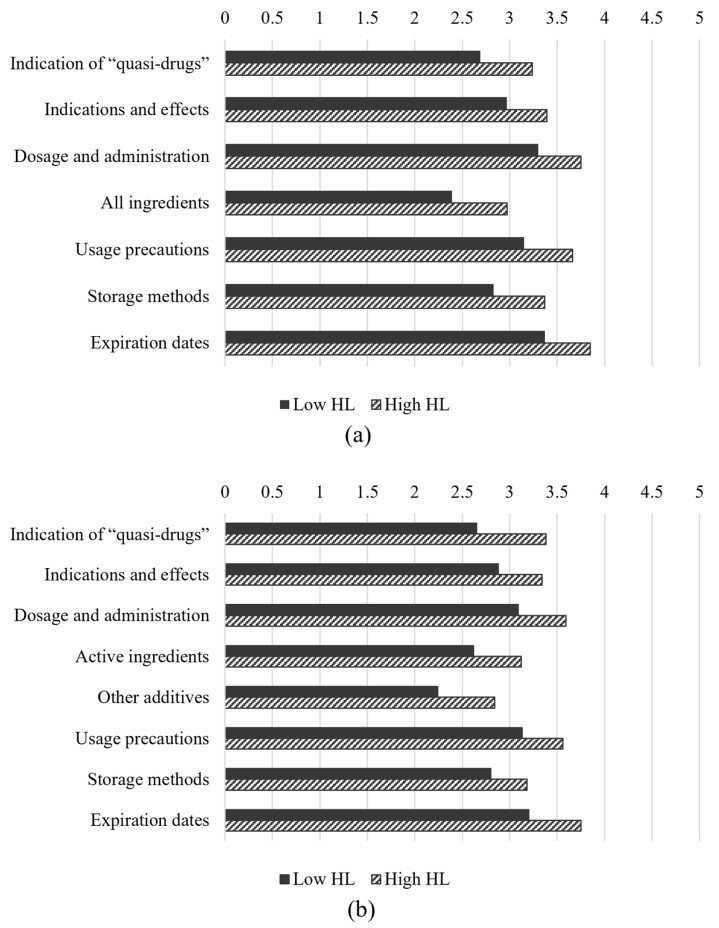
Label-reading habits affecting the participants’ health literacy levels. (**a**) Degree of reading mask labels; (**b**) degree of reading hand sanitizer labels. HL, health literacy. The degree of reading mask and hand sanitizer labels was assessed using a 5-point Likert scale, where 1 indicated “never read” and 5 indicated “read it all”. All *p*-values were <0.001.

The study population was divided into two groups based on their HL levels, with the majority (68.4%) falling into the low-HL category. Figure 1 presents the analysis of the label-reading behaviors for masks and hand sanitizers, stratified according to the HL levels. The degree of label reading was assessed using a 5-point Likert scale, where 1 indicated “never read” and 5 indicated “read it all”. Across all categories, participants with high HL consistently demonstrated more thorough label reading than those with low HL. For instance, the mean score for reading mask indications was 3.75 (SD = 1.27) for the high-HL group, compared to 3.30 (SD = 1.20) for the low-HL group (*p* < 0.001). Similar trends were observed for hand sanitizer labels, where high-HL participants reported more thorough reading across all categories (*p* < 0.001).

**Figure 2:**
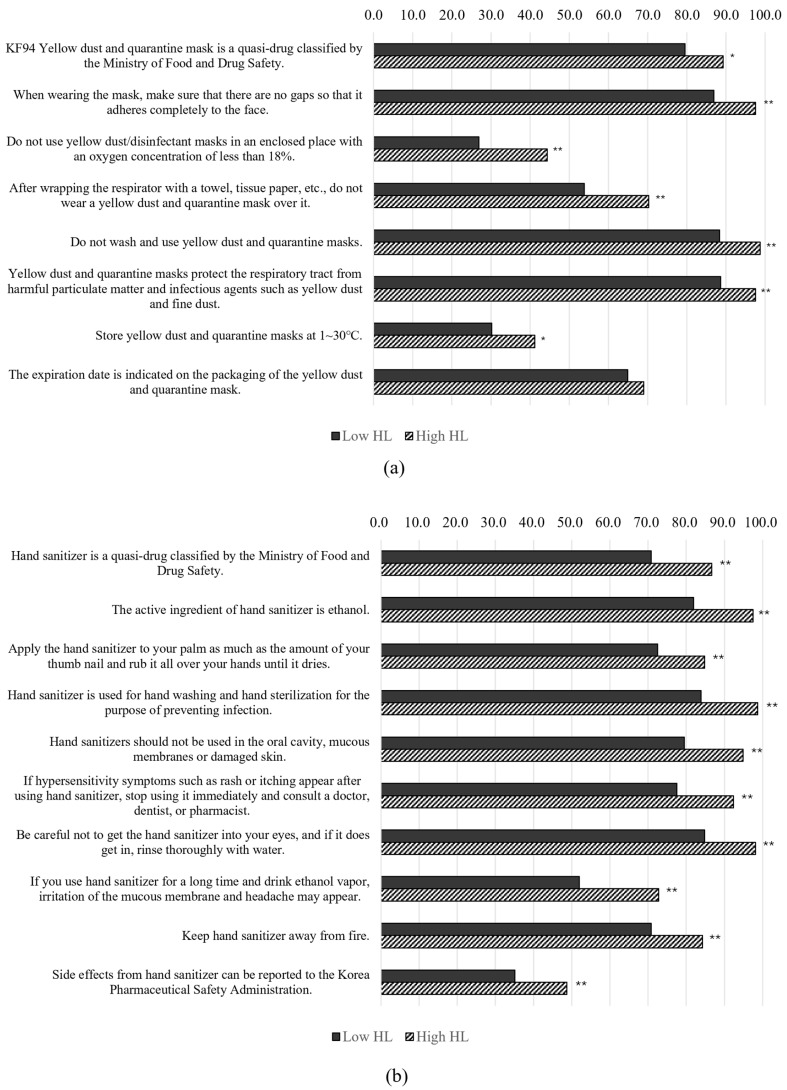
(**a**) Percentage of respondents reporting preexisting knowledge of mask use, stratified according to HL level. (**b**) Percentage of respondents reporting preexisting knowledge of hand sanitizer use, stratified according to HL level. HL, health literacy. * *p* < 0.05, ** *p* < 0.01.

Figure 2 illustrates that there were significant differences in mask and hand sanitizer knowledge between participants with low and high HL levels. For mask-related knowledge, a significantly larger proportion of high-HL participants (89.2%) recognized the KF94 yellow dust and quarantine mask as a quasi-drug, compared to 79.5% of those with low HL (*p* = 0.011). Moreover, high-HL respondents demonstrated better knowledge of proper mask usage, with a greater percentage understanding that masks should adhere completely to the face (97.5% vs. 86.8%, *p* < 0.001) and should not be used in enclosed spaces with low oxygen (O_2_) concentrations (44.3% vs. 26.9%, *p* < 0.001). Similar trends were observed for hand sanitizer-related knowledge. High-HL respondents were more likely to identify hand sanitizer as a quasi-drug (86.7% vs. 70.8%, *p* < 0.001), recognize ethanol as the active ingredient (97.5% vs. 81.9%, *p* < 0.001), and understand that hand sanitizers should not be applied on damaged skin (94.9% vs. 79.5%, *p* < 0.001). The internal consistency of the 18 questions assessing the knowledge of mask and hand sanitizer use was measured using Cronbach’s alpha, which ranged from 0.816 to 0.830, indicating good reliability.

The multivariable logistic regression analysis identified HL as a significant factor influencing the correct knowledge of mask and hand sanitizer use. For mask-related knowledge, participants with high HL demonstrated a substantially higher likelihood of correctly identifying important information (OR 2.56, 95% CI: 1.69–3.89) (Table 2). This association was even more pronounced for hand sanitizer-related knowledge, with high-HL participants showing a markedly increased probability of possessing correct knowledge (OR 5.39, 95% CI: 3.31–8.77) (Table 3). Notably, other demographic factors, such as the participants’ age, gender, education level, monthly income, employment status, marital status, or household size, were not significantly correlated with their knowledge levels regarding either masks or hand sanitizers. This lack of association was consistent across both product categories, underscoring HL’s unique and pivotal role in determining knowledge of the proper use of these preventive health products.

## 4. Discussion

This study examined the relationship between HL and consumers’ understanding of mask and hand sanitizer labels in the post-COVID-19 pandemic context. The findings revealed that there were significant differences in label comprehension and knowledge between individuals with high and low HL levels. Specifically, participants with higher HL demonstrated better recognition of these products’ “quasi-drug” designation and a more comprehensive understanding of the efficacy, usage instructions, ingredients, usage precautions, storage methods, and expiration dates of both masks and hand sanitizers.

These results are consistent with previous research demonstrating that higher HL is strongly associated with increased engagement in preventive health behaviors and improved comprehension of health-related materials [11,22,23]. This study highlights the crucial role of HL in public health communication and education. Individuals with higher HL demonstrated a greater ability to understand and apply health information, such as information about correct mask usage and hand sanitization practices [4,24,25]. For example, the enhanced understanding observed among the high-HL participants in this study facilitated more effective disease prevention and control. Significant gaps in understanding the proper usage of hand sanitizers and masks were evident between the high- and low-HL groups [5,26,27].

Furthermore, this study aligns with broader findings linking HL with better health outcomes and preventive health behaviors. By focusing on the specific context of quasi-drug labels in the post-pandemic era, this study reinforces the importance of public health strategies that prioritize enhancing HL to foster improved health engagement. Sørensen et al. (2012) noted that simplifying health-related messages significantly boosts comprehension and health engagement, especially among populations with low HL [9]. These findings emphasize the value of tailored communication strategies to address literacy disparities and promote equitable health outcomes.

One of the most notable findings of this study is the significant variation in label-reading behaviors between individuals with high and low HL. Participants with higher HL consistently demonstrated more thorough engagement with the product labels. For example, high-HL individuals reported reading critical sections of the labels—such as the usage precautions, active ingredients, and expiration dates—more closely than their low-HL counterparts. This supports earlier findings presented by Wolf et al. (2006), who observed that individuals with higher HL were more thorough and accurate in processing prescription drug warning labels—a pattern that was similarly observed in this study regarding the labels of masks and hand sanitizers [17]. This pattern suggests that individuals with higher HL are more proactive in seeking and processing health information, a behavior that likely enhances their ability to make informed decisions regarding the safe and effective use of health products.

The difference in label engagement can be attributed to the cognitive and motivational advantages associated with higher HL. These individuals often have the skills and confidence to navigate complex health information, a concept that is supported by previous studies demonstrating that higher HL fosters self-efficacy and better comprehension of medical instructions [10,17]. In contrast, individuals with lower HL frequently report feeling overwhelmed by detailed labels, which may lead to disengagement or the misuse of products. These challenges underscore the necessity of tailoring public health messages to accommodate varying literacy levels [28].

The decision to stratify the sociodemographic characteristics according to gender was driven by the hypothesis that gender may influence health-related behaviors and literacy levels, as supported by prior research showing that men and women often exhibit distinct patterns in terms of health communication and preventive practices [29,30]. The findings revealed that, despite reporting lower income levels and higher rates of part-time employment or unemployment, women demonstrated higher HL scores compared to men. This result is noteworthy as it suggests that socioeconomic disparities may not directly translate into differences in HL, highlighting the need for further research to identify the underlying factors contributing to these variations.

Understanding the disparities in label-reading behaviors has critical implications for public health strategies. The substantial disparities between the high- and low-HL groups suggest a need for targeted educational interventions. Public health campaigns should consider simplifying complex information and employing more accessible language to reach individuals with lower HL. Tools such as visual aids, digital platforms, and practical demonstrations have proven effective in enhancing comprehension among low-HL groups [28,31,32]. Additionally, interventions could focus on increasing the motivation and self-efficacy of these individuals to enable them to engage more thoroughly with health-related information. For those with higher HL, providing comprehensive and detailed content can further enhance their informed decision-making capabilities.

This study was affected by certain limitations that must be acknowledged. First, the cross-sectional design limited the capacity to establish causal relationships between HL and label comprehension, necessitating longitudinal studies for such inferences. Second, the reliance on self-reported data may have introduced response biases, such as social desirability bias, potentially leading to the participants’ overreporting of their understanding of health information. Third, the HL assessment was based on a specific scale, and alternative measures of HL might yield varying results. Fourth, despite efforts to recruit a representative sample through scientific methods considering the population structure, the use of an online survey may have excluded individuals who were not familiar with smart devices. Lastly, the exclusion of individuals aged 70 and above may have limited the study’s ability to capture the literacy levels among the elderly population.

Future research should explore the effectiveness of diverse educational strategies and communication methods in improving HL and label comprehension. Interventions tailored to specific demographic groups, such as older adults or individuals with a lower socioeconomic status, could be particularly beneficial. Moreover, examining the role of digital HL in the context of online health information and resources would be valuable, especially as the use of digital platforms continues to rise [33,34]. Further investigation of the long-term impact of enhanced HL on health behaviors and outcomes is also warranted.

## 5. Conclusions

In conclusion, this study highlights the critical role of HL in improving consumers’ comprehension of mask and hand sanitizer labels in the post-pandemic context. The findings demonstrate that higher HL significantly enhances individuals’ understanding and interpretation of quasi-drug classifications, usage instructions, and safety precautions, emphasizing the importance of addressing literacy gaps in public health initiatives. Notably, this study found that women generally exhibited higher HL scores despite their socioeconomic disadvantages, underscoring the need for targeted educational strategies to support vulnerable populations. By simplifying health information, utilizing visual aids, and enhancing communication strategies, public health authorities can bridge the gaps in HL, fostering informed decision-making and safer health practices during future public health emergencies.

## Figures and Tables

**Table 1:** Demographic characteristics of the study population (*N* = 500).

Variable	Men		Women		Total		*p*-Value
	(*N* = 254)		(*N* = 246)		(*N* = 500)		
	*N*	(%)	*N*	(%)	*N*	(%)	
Age (years)							
20–39	89	(35.0)	80	(32.5)	169	(33.8)	0.797
40–59	114	(44.9)	112	(45.5)	226	(45.2)	
≥60	51	(20.1)	54	(22.0)	105	(21.0)	
Mean ± SD	45.2	±13.6	46.1	±13.7	45.7	±13.6	0.458
Marital status							
Single	106	(41.7)	105	(42.7)	211	(42.2)	0.901
Married	148	(58.3)	141	(57.3)	289	(57.8)	
Household size							
1	48	(18.9)	39	(15.9)	87	(17.4)	0.164
2	44	(17.3)	59	(24.0)	103	(20.6)	
≥3	162	(63.8)	148	(60.2)	310	(62.0)	
Education level							
High school or lower	36	(14.2)	57	(23.2)	93	(18.6)	0.028
University	190	(74.8)	169	(68.7)	359	(71.8)	
Graduate school	28	(11.0)	20	(8.1)	48	(9.6)	
Monthly income (USD)							
<722	25	(9.8)	47	(19.1)	72	(14.4)	<0.001
722–2166	54	(21.3)	74	(30.1)	128	(25.6)	
2167–3610	86	(33.9)	64	(26.0)	150	(30.0)	
3611–5055	68	(26.8)	36	(14.6)	104	(20.8)	
≥5056	21	(8.3)	25	(10.2)	46	(9.2)	
Employment status							
Full-time	187	(73.6)	100	(40.7)	287	(57.4)	<0.001
Part-time	16	(6.3)	39	(15.9)	55	(11.0)	
Unemployed	51	(20.1)	107	(43.5)	158	(31.6)	
Health literacy							
Low	180	(70.9)	162	(65.9)	342	(68.4)	0.001
High	74	(29.1)	84	(34.1)	158	(31.6)	
Mean ± SD	42.0	23.2	48.3	19.4	45.1	21.6	0.001

SD, standard deviation; USD, United States dollars.

**Table 2:** Factors influencing comprehensive knowledge of mask use.

Variable	Total	Respondents with Comprehensive Knowledge		Crude Odds Ratio		Adjusted Odds Ratio	
	(*N*)	(*N*, %)		(OR, 95% CI)		(OR, 95% CI)	
Age (years)							
20–39	169	46	(27.2)	1.00		1.00	
40–59	226	63	(27.9)	1.03	(0.66–1.62)	0.89	(0.53–1.49)
≥60	105	30	(28.6)	1.07	(0.62–1.84)	0.85	(0.44–1.63)
Gender							
Men	246	67	(27.2)	1.00		1.00	
Women	254	72	(28.3)	1.06	(0.72–1.56)	1.18	(0.76–1.82)
Health literacy							
Low	342	74	(21.6)	1.00		1.00	
High	158	65	(41.1)	2.53	(1.68–3.81)	2.56	(1.69–3.89)
Education level							
High school or lower	93	22	(23.7)	1.00		1.00	
≥University	407	117	(28.7)	1.30	(0.77–2.20)	1.38	(0.79–2.41)
Monthly income (USD)							
<2167	200	58	(29.0)	1.00		1.00	
≥2167	300	81	(27.0)	0.91	(0.61–1.35)	0.77	(0.48–1.24)
Employment status							
Full-time	287	77	(26.8)	1.00		1.00	
Part-time	55	13	(23.6)	0.84	(0.43–1.66)	0.90	(0.44–1.88)
Unemployed	158	49	(31.0)	1.23	(0.80–1.88)	1.25	(0.76–2.06)
Marital status							
Single	211	54	(25.6)	1.00		1.00	
Married	289	85	(29.4)	1.21	(0.81–1.81)	1.41	(0.79–2.52)
Household size							
1	87	24	(27.6)	1.00		1.00	
2	103	28	(27.2)	0.98	(0.52–1.86)	0.73	(0.34–1.58)
≥3	310	87	(28.1)	1.02	(0.60–1.74)	0.79	(0.41–1.53)

OR, odds ratio; CI, confidence interval; USD, United States dollars.

**Table 3:** Factors influencing comprehensive knowledge of hand sanitizer use.

Variable	Total	Respondents with Comprehensive Knowledge		Crude Odds Ratio		Adjusted Odds Ratio	
	(*N*)	(*N*, %)		(OR, 95% CI)		(OR, 95% CI)	
Age (years)							
20–39	169	92	(54.4)	1.00		1.00	
40–59	226	144	(63.7)	1.47	(0.98–2.21)	1.29	(0.79–2.11)
≥60	105	65	(61.9)	1.36	(0.83–2.24)	1.05	(0.56–1.96)
Gender							
Men	246	150	(61.0)	1.00		1.00	
Women	254	151	(59.4)	0.94	(0.66–1.34)	0.95	(0.63–1.43)
Health literacy							
Low	342	168	(49.1)	1.00		1.00	
High	158	133	(84.2)	5.51	(3.42–8.88)	5.39	(3.31–8.77)
Education level							
High school or lower	93	49	(52.7)	1.00		1.00	
≥University	407	252	(61.9)	1.46	(0.93–2.30)	1.57	(0.94–2.61)
Monthly income (USD)							
<2167	200	116	(58.0)	1.00		1.00	
≥2167	300	185	(61.7)	1.17	(0.81–1.68)	0.94	(0.60–1.47)
Employment status							
Full-time	287	174	(60.6)	1.00		1.00	
Part-time	55	24	(43.6)	0.50	(0.28–0.90)	0.53	(0.27–1.03)
Unemployed	158	103	(65.2)	1.22	(0.81–1.82)	1.27	(0.79–2.05)
Marital status							
Single	211	119	(56.4)	1.00		1.00	
Married	289	182	(63.0)	1.32	(0.92–1.89)	1.24	(0.72–2.14)
Household size							
1	87	54	(62.1)	1.00		1.00	
2	103	64	(62.1)	1.00	(0.56–1.81)	0.69	(0.34–1.42)
≥3	310	183	(59.0)	0.88	(0.54–1.44)	0.64	(0.35–1.19)

OR, odds ratio; CI, confidence interval; USD, United States dollars.

## Data Availability

The data that support the findings of this study are available from the corresponding author upon reasonable request.
